# Examining the safety of colon anastomosis on a rat model of ischemia-reperfusion injury

**DOI:** 10.1186/1749-7922-8-24

**Published:** 2013-07-02

**Authors:** David Czeiger, Anton Osyntsov, Lidia Osyntsov, Chad G Ball, Roy Gigi, Gad Shaked

**Affiliations:** 1 Department of General Surgery and Trauma Unit, Soroka University Medical Center and Ben-Gurion University, Beer Sheva, Israel; 2 Department of General Surgery, Soroka University Medical Center and Ben-Gurion University, Beer Sheva 84101, Israel; 3 Pathology Institute, Soroka University Medical Center and Ben- Gurion University, Beer Sheva, Israel; 4 Department of Surgery, University of Calgary, Calgary, Alberta, Canada; 5 Department of Orthopedics, Tel Aviv Sourasky Medical Center, Tel Aviv, Israel

**Keywords:** Ischemia-reperfusion, Injury, Colon, Intestinal anastomosis, Rat model, Emergency surgery

## Abstract

**Introduction:**

Intestinal ischemia and reperfusion can impair anastomotic strength.

The purpose of this study was to evaluate the safety of delayed colon anastomosis following remote ischemia-reperfusion (IR) injury.

**Methods:**

Rats divided into two groups underwent bilateral groin incisions, however only the study group had femoral artery clamping to inflict IR injury. Twenty-four hours following this insult, the animals underwent laparotomy, incision of the transverse colon and reanastomosis. End points included anastomotic leakage, strength and histopathological features.

**Results:**

Anastomotic leak among IR animals (22.2%) was not statistically different in comparison to the controls [10.5% (p = 0.40)]. Anastomotic mean burst pressures showed no statistically significant difference [150.6 ± 15.57 mmHg in the control group vs. 159.9 ± 9.88 mmHg in the IR group (p = 0.64)]. The acute inflammatory process in the IR group was similar to controls (p = 0.26), as was the chronic repair process (p = 0.88). There was no significant difference between the inflammation:repair ratios amongst the two groups (p = 0.67).

**Conclusion:**

Primary colon repair is safe when performed 24 hours following systemic IR injury.

## Introduction

Ischemia-reperfusion (IR) injury represents a fundamental common pathway of tissue damage in a wide variety of disease and surgical processes such as major trauma, acute mesenteric ischemia, septic and hypovolemic shock, abdominal aortic aneurism surgery, and cardiopulmonary bypass [1,2]. Interruption of blood supply results in ischemic injury to all body systems and especially to high metabolically active tissues; the intestine is a prominent example of a sensitive tissue to IR injury which is associated with high morbidity and mortality [1]. Paradoxically, restoration of blood flow to the ischemic tissue augments cell injury by delivering toxic mediators induced in the ischemic tissue into the circulation thus affecting distant organs. This might lead to the development of systemic inflammatory response syndrome, which can progress to multiple organ failure and death [2]. Among the toxic mediators produced in the IR injured tissue are acute-phase proteins, pro-inflammatory cytokines, oxygen free radicals, and components of the complement system [3]. Emergency surgery and trauma situations may require abbreviated procedures during the initial phase of shock and organ ischemia. Definitive procedures including anastomosis to restore bowel continuity are undertaken 24 hours or more afterward. Two common examples of such situations are the strategy of damage control surgery in seriously injured patients, and acute mesenteric ischemia. In damage control laparotomy the goal in the emergency surgery is to stop bleeding and to control spillage from the intestine. In the second operation, which is done after the patient’s deranged physiology is corrected, bowel anastomosis may be created. In mesenteric ischemia gangrenous segments of the bowel are resected, while fluid resuscitation continues. Not infrequently, the patient condition does not allow performing primary anastomosis. This may be done during a second look operation 24 hours later. The spectrum of the effects of IR injury on the intestine is broad and ranges from a transient absorptive impair following mucosal damage to frank gangrene of the bowel [4]. Previous reports have shown that ischemia and reperfusion of the intestinal wall can lead to impaired anastomotic strength [5-8]. However, there is not enough evidence in the literature to show the safety of delayed bowel anastomosis following systemic IR injury. We hypothesized that IR injury would adversely affect the safety of colonic anastomoses performed 24 hours following the injury. To evaluate this hypothesis we investigated the effects of IR injury on the healing of colon anastomoses in a rat model.

## Materials and methods

The protocol employed in this study was approved by the Committee for the Ethical Care and Use of Laboratory Animals of the Ben-Gurion University of the Negev (approval code IL-41-7-2006). It included a provision that any rat exhibiting evidence of distress (such as restlessness or aggressive behavior) be immediately euthanized. Rats were acclimated to the laboratory for 2 weeks prior to the study and had free access to water and food at all times. A total of 40 male Sprague–Dawley rats (average weight 350 g) were used. The number of animals in each group was considered satisfactory based on a two-sided sample size determination (power analysis), assuming power of 0.80 and significance of 0.05. All rats were anesthetized with inhaled isoflurane 1% at a rate of 3–5 L/min. The study group (n = 20) underwent bilateral groin incision and clamping the femoral arteries for 30 minutes. The control group (n = 20) had a similar sham operation without inducing extremities ischemia. All wounds were then sutured with 4/0 silk. Twenty-four hours following this insult, all animals were anesthetized and underwent a midline laparotomy, full circumference incision of the transverse colon (including resection of 0.5 cm of mesentery on each side of the colon) and reanastomosis (end-to-end) using 4/0 polyglycolic acid sutures. The animals were then followed up and sacrificed one week later. The peritoneal cavity was subsequently explored for the presence of perforation, and local or generalized peritonitis.

Anastomotic healing was assessed by determining anastomotic burst pressures, as well as by formal histopathological examination. The transverse colon was dissected free of adhesions and resected. One end of this segment was ligated, and a catheter connected to a sphygmomanometer was secured to the other end. Air was then pumped into the segment of colon, which was submerged in water. Intraluminal pressure was monitored continuously while the air was injected. The intraluminal pressure at which air leakage from the anastomosis occurred was recorded as the burst pressure. More specifically, this parameter represents the mechanical strength of the anastomosis. The colon specimen was then processed and evaluated for three parameters of acute inflammation: (1) total number of polymorphonuclear cells, (2) total number of red blood cells and (3) the amount of fibrin, as well as for three parameters of chronic wound healing: (1) total number of mononuclear cells, (2) total number of fibroblasts and (3) total amount of collagen. Each parameter was graded from 0 to 4. The colon surgeon and the pathologist were each blinded with regard to the individual group allocation history of the animals. Statistical analysis was performed using GraphPad Prism version 4.00 for Windows, GraphPad Software, San Diego, California, USA. Parametric results are expressed as mean ± SEM and were compared using an unpaired t-test. Two-tailed p < 0.05 was considered as having a statistical significance.

## Results

**Figure 1:**
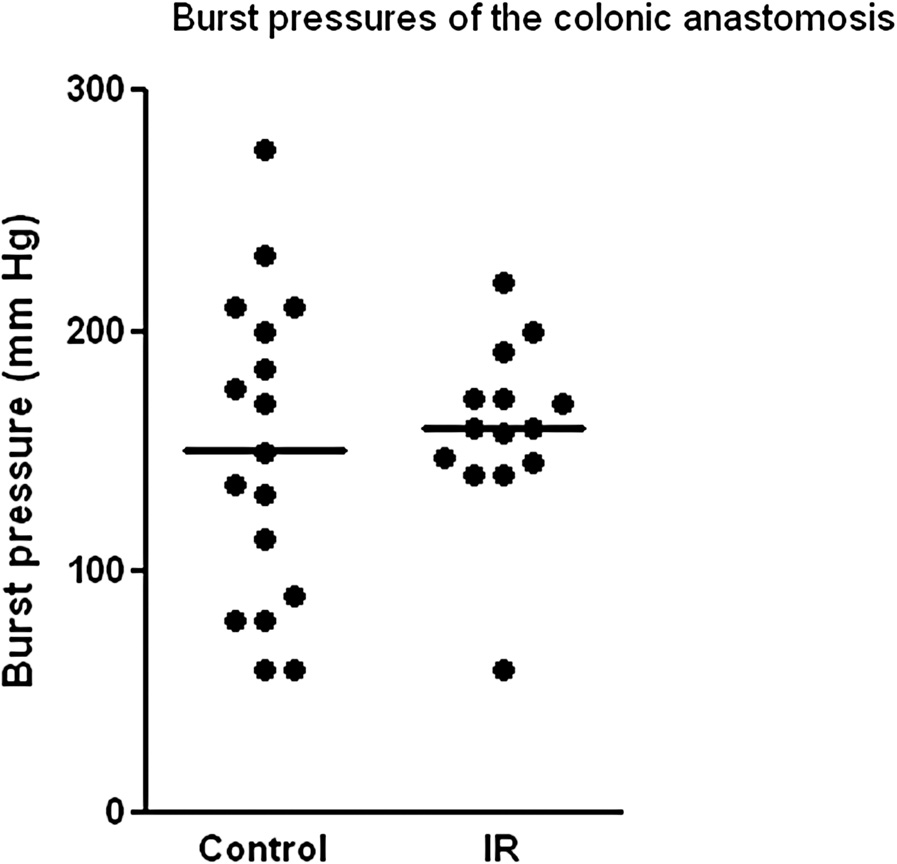
**Colon anastomotic strength is reflected by burst pressure expressed by mmHg.** Individual values and means are shown for the IR and control groups. The variance of distribution of burst pressures around the mean pressure is significantly smaller in the IR group compared to the control group (p = 0.025).

Three animals were excluded from the study because they died before the completion of the surgical procedure (1 control and 2 IR). One rat in the IR group also died during the 7-day follow-up period (p > 0.05). Autopsy of this animal revealed an anastomotic leak and diffuse peritonitis. Among the animals that completed the follow-up period, anastomotic leak and a severe peritoneal reaction was observed in 3 animals within the IR cohort, and in 2 control animals. The anastomotic leak rate among IR animals (22.2%) was not statistically different in comparison to the controls [10.5% (p = 0.40)]. The anastomotic mean burst pressures also showed no statistically significant difference [150.6 ± 15.57 mmHg in the control group vs. 159.9 ± 9.88 mmHg in the IR group (p = 0.64)]. The specific distribution of individual burst pressures is displayed in Figure 1. More specifically, the burst pressures among the IR group display significantly less variance than the control group. The F test used to compare variances shows a significant difference (p = 0.025). To statistically compare histopathological results, 3 grades were assigned for both the inflammatory process and chronic repair process for each animal. Student’s t-test comparing the means of sums and Fisher’s exact test comparing inflammation:repair ratios of the two groups revealed no significant statistical differences. The acute inflammatory process in the IR group was similar to controls (p = 0.26), as was the chronic repair process (p = 0.88). There was also no significant difference between the inflammation:repair ratios in the two groups (p = 0.67).

## Discussion

The goal of this study was to examine the safety of colon anastomosis performed 24 hours after profound systemic ischemia-reperfusion injury. Our results show that resection and anastomosis of the colon is safe, when performed twenty-four hours following profound, remote IR injury. This is based on similar mortality and anastomotic leak ratios (although a non-significant trend towards a higher incidence of anastomotic leak among the IR animals was noted), comparable anastomotic mechanical strengths, and equivalent histological features of the anastomosis between the IR and the control groups.

Today, in 2013, anastomotic leak after colorectal resection still has lethality of 6-22% and morbidity leading to reoperation and permanent stoma in 56% [9]. There is convincing evidence in the literature that primary repair or anastomosis is appropriate for the management of most colonic injuries and for other emergent surgical situations [10-17]. In contrast, there is little methodologically sound evidence outlining the outcome of a colon anastomosis in the setup of severe IR. Damage control surgery (DCS) is probably one of the most common situations where the surgeon faces the dilemma of creating colonic anastomosis in a delayed fashion after IR injury. Clinical retrospective series have revealed contradictory conclusions regarding the safety of this procedure. Miller et al. [18] concluded that delayed anastomoses in patients undergoing DCS is safe, whereas Weinberg and colleagues reported a significant colon related complication rate in patients who were treated by resection and anastomosis [19]. A third group also identified a higher incidence of colonic anastomotic leakage among DCS patients who had resection followed by anastomosis; however they declared that resection and anastomosis is still considered safe [20]. Ott pointed in a recently published manuscript that colon anastomosis is safe unless the abdomen remains open. He also regards the left colon as more vulnerable to leak under these conditions [21]. It is obvious that limitations in these studies include heterogeneous patient populations, variance in patients’ clinical condition and surgeons’ preference, and even the very definition of DCS by different surgeons.

To overcome these limitations inherent in clinical retrospective studies we created a rat model of IR injury followed by resection and reansatomosis of the transverse colon. IR injury has been intensively investigated since the 1970s. The IR phenomenon represents the common underlying pathophysiological process to a variety of medical conditions and surgical procedures. Tissue ischemia with inadequate oxygen supply followed by successful reperfusion initiates a wide and complex array of inflammatory responses that may both aggravate local injury, as well as induce impairment of remote organ function [22]. Review of the literature reveals experimental studies evaluating the effect of transient preoperative IR on gut anastomotic strength [6,8,23-29]. The results of these studies were equivocal. This may partially be explained by the degree and duration of the inflicted ischemia [26]. Also, the surgical procedure was done on small bowel in some of the studies [30], while in others large bowel anastomoses were tested [31]. Furthermore, in the previous models the ischemia was done by clamping the blood supply of the resected segment of intestine, and/or performed the intestinal anastomosis immediately following the IR injury. Kuzu et al. attempted to demonstrate the systemic nature of IR by occluding the superior mesenteric artery and its collaterals and immediately thereafter they resected and reansatomose the left colon [7]. Posma described the effect of a prolonged interval between IR and anastomotic construction on the anastomosis healing, but used a model of local mesenteric ischemia [26].

We believe that the present model, with severe systemic remote ischemia, performance of a colon anastomosis 24 hours later, and testing the anastomotic strength after one week, more closely resembles the true conditions of some emergent conditions that the surgical approach for them is still uncertain. Several mechanisms have been suggested to explain the blunting of the IR deleterious effect on bowel anastomoses when these are constructed late after the insult. One is subsidence of the harmful effects over the time elapsed from the insult to the creation of the anastomosis. Another explanation is the protective effect of ischemic preconditioning [30,32]. Recently, studies have been published on prevention/alleviation the effect of IR injury by inhibiting compliment system activation [33], by applying antioxidants [34,35], and trace elements [36]. Another trend for attenuating effects of IR injury is ischemic postconditioning [37-39].

In our experiment we amplified the local ischemia at the site of anastomosis by resecting 0.5 cm of mesentery on each side of the divided transverse colon. Even under these stringent conditions we did not observe the expected IR harmful effects. On the other hand, our results showed no benefit to the ischemic group. This should question the protective effect of ischemic preconditioning in this setup.

In summary, this rat model augments the literature which support delayed primary repair after ischemia-reperfusion injury. However, more laboratory and clinical evidence is required before final conclusion can be drawn. More studies are also needed to understand the attenuation of the harmful effects of IR on intestinal anastomosis when performed 24 hours after the injury.

## Competing interests

The authors declare that they have no competing interests.

## Authors’ contributions

DC participated in the design of the study, performed the statistical analysis, and revised the manuscript, AO carried out the operations, LO performed the pathological examinations and the evaluations of the specimens, CB was involved in drafting the manuscript and revising it critically, RG participated in the laboratory work and animal assays, GS initiate the study, created its design and wrote the manuscript. All authors read and approved the final manuscript.
